# Assessing the reproducibility of American College of Surgeons National Surgical Quality Improvement Program (ACS-NSQIP) arthroplasty studies

**DOI:** 10.1186/s13018-025-05538-0

**Published:** 2025-03-01

**Authors:** Ayobami S. Ogunsola, Michael C. Marinier, Arman C. Hlas, Jacob M. Elkins

**Affiliations:** Department of Orthopedics and Rehabilitation, University of Iowa, 200 Hawkins Drive, Iowa City, IA 52242 USA

**Keywords:** Reproducibility, Arthroplasty, ACS-NSQIP, Smoking, Risk factors, Surgical outcomes

## Abstract

**Background:**

Utilization of large-volume clinical registries for observational research has gained popularity in orthopaedic literature. However, concerns exist regarding inadequate reporting of methodology in this type of research. Despite these concerns, the reproducibility of such studies has not been adequately assessed in existing literature. This study aims to assess the reproducibility of American College of Surgeons National Surgical Quality Improvement Program (ACS-NSQIP) arthroplasty studies on smoking as a risk factor for poor surgical outcomes by employing identical datasets and statistical methods.

**Methods:**

A systematic PubMed search between 2013 and 2023 identified ACS-NSQIP studies involving hip or knee arthroplasty and smoking as a potential risk factor for poor surgical outcomes. Each study’s methods were reproduced by a trained statistician based on the reported methodology. In cases where certain steps were not explicitly stated, the statistician made informed decisions to reproduce those steps. Adjusted odds ratios (aORs) and *p*-values (α = 0.05) were compared between the original and reanalyzed datasets.

**Results:**

The initial search yielded 43 studies, with 11 meeting inclusion criteria resulting in the reanalysis of 268 aORs. Upon reanalysis, 12.69% of the original studies’ aORs changed in interpretation, while 13.43% experienced a change in statistical significance. The average magnitude change of each aOR across all studies was 17.22%, and the sample size (N) in reanalysis varied by up to 47.84%. Among the 11 commonly cited studies, approximately one in eight objective conclusions changed in interpretation or statistical significance.

**Conclusion:**

Inconsistent reproducibility exists across many arthroplasty studies that utilize the ACS-NSQIP database. These findings highlight the importance of rigorous reporting of study methodology, data collection, and statistical analyses when utilizing large-volume databases in orthopaedic research. This burden of responsibility should be shared among authors, peer reviewers, and orthopaedic journals to confirm the accuracy and validity of published database research.

**Level of evidence:**

This study systematically reviewed and analyzed, in attempt to reproduce, published arthroplasty studies utilizing ACS-NSQIP database to assess smoking as a potential risk factor for poor surgical outcomes. All analyzed studies included Level III Evidence, therefore this current study compares reproduced Level III Evidence to the original Level III Evidence.

**Supplementary Information:**

The online version contains supplementary material available at 10.1186/s13018-025-05538-0.

## Introduction

In the past decade, utilization of large-volume clinical registries for observational research has increased markedly in orthopaedic literature [1]. These national databases offer a vast and accessible study population with numerous variables available for investigation over a designated time period [2]. Although randomized control trials remain the gold standard in orthopaedic research, such trials can be expensive, time-consuming, and limited in sample size to enable generalizability to broader populations [3, 4]. Some research topics may also be ill-suited for these designs owing to ethical concerns or lack of feasibility in answering specific questions in today’s era [1, 3]. Consequently, well-conducted observational studies have emerged as a viable alternative to answer research questions that are too difficult or costly to address through other study designs [1]. Large-volume clinical registries provide robust data to conduct these studies in a low-cost and efficient manner.

**Fig. 1:**
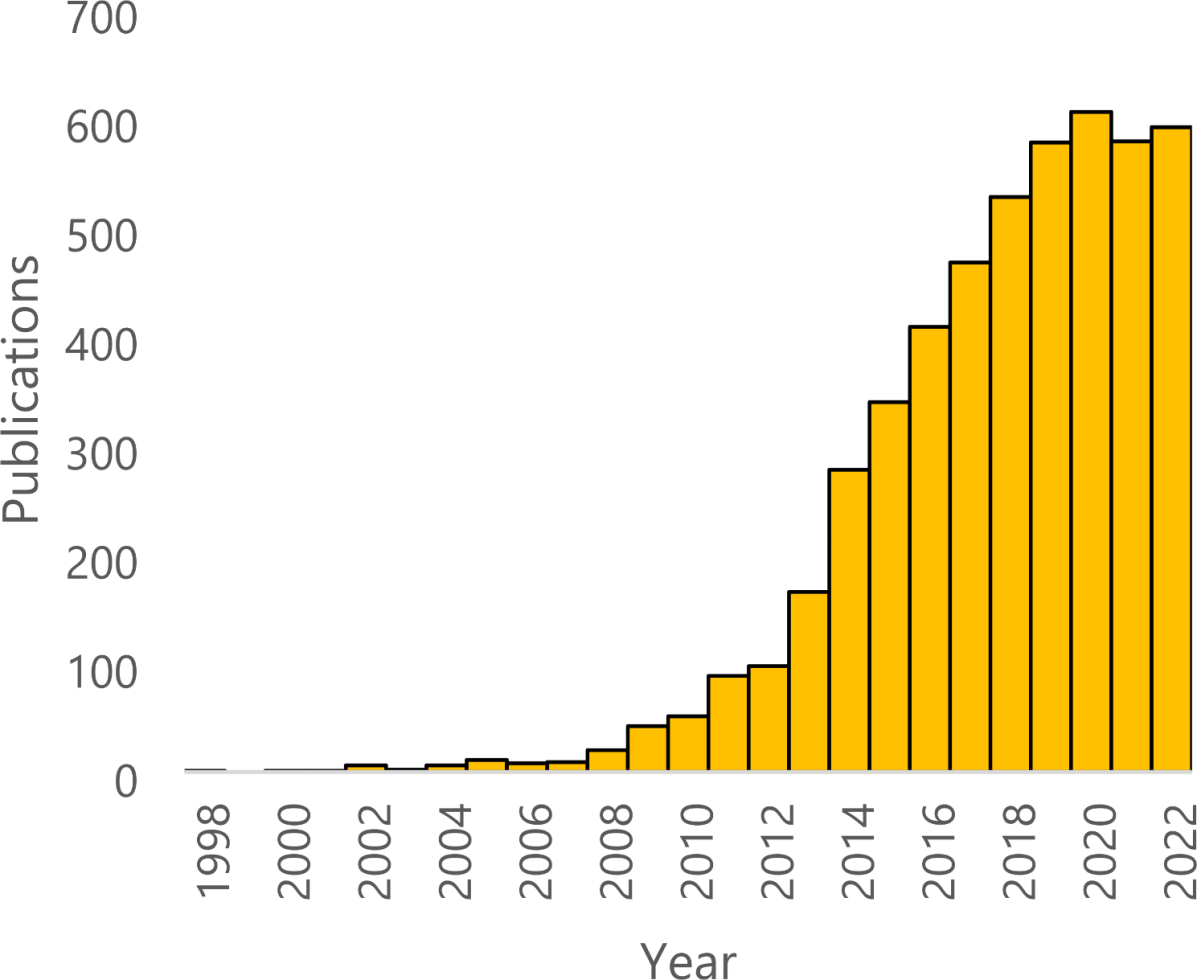
ACS-NSQIP database publication count, 1998–2022

The American College of Surgeons National Surgical Quality Improvement Program (ACS-NSQIP) database remains one of the most commonly utilized clinical registries in surgical research (Fig. 1). This database contains preoperative, intraoperative, and 30-day postoperative data prospectively collected by clinical reviewers through a standardized process with frequent data auditing [5, 6]; as a result, the strength of NSQIP lies in the accuracy of collected data and its utility as a resource both developed and validated by surgeons [3, 6]. In orthopaedics, many authors have utilized ACS-NSQIP in arthroplasty research to identify risk factors for poor surgical outcomes including postoperative complications, increased length of hospital stay, and hospital readmission. The effects of smoking as a potential risk factor have been frequently explored in much of this research [7–17]. These studies largely report Level III evidence due to their observational design [18].

Although observational database research represents a growing subset of orthopaedic literature, several limitations exist with this study design. Observational studies can only establish association between predictor and outcome variables, and cannot be used to determine causality [19]. Lack of control over predictor variables introduces the potential for bias or confounding in these studies [20]. Extensive data mining to unveil statistically significant associations can also lead to poor recollection and reporting of study methodology. Recent studies have demonstrated that the majority of database research fails to adhere to current methodologic reporting standards [21, 22], which can lead to further decreases in study validity. One way to confirm the reliability of such research is by assessing study reproducibility [23, 24]. While observational database research offers the opportunity to address questions that are difficult to answer through other study designs, the reproducibility of such studies has not been adequately assessed in existing literature.

This study aimed to determine the reproducibility of ACS-NSQIP hip or knee arthroplasty studies assessing smoking as a risk factor for poor surgical outcomes by employing identical datasets and statistical methods as described in selected publications.

## Methods

### Search strategy and study selection

**Fig. 2:**
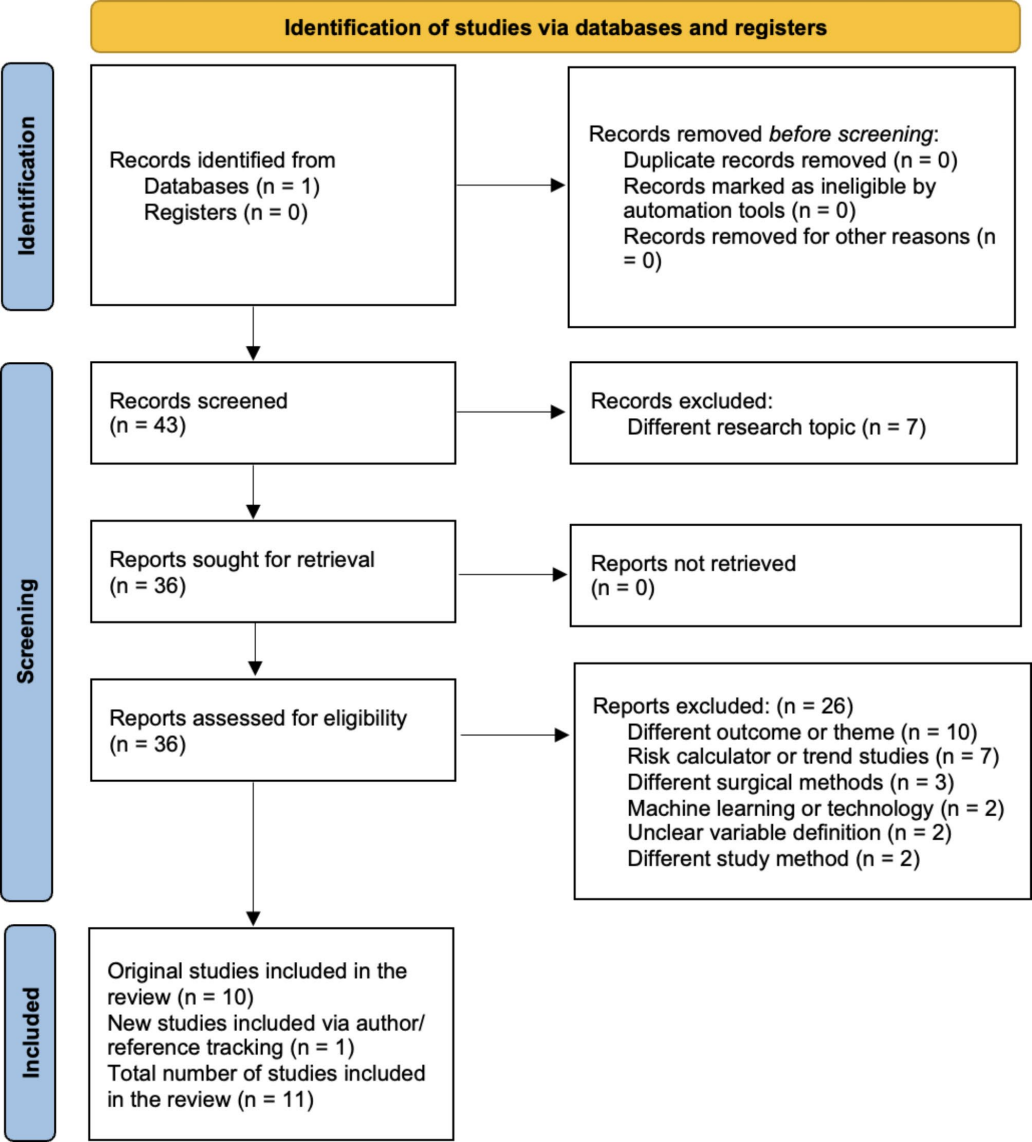
Flow diagram for study selection and inclusion in ACS-NSQIP reproducibility study

A PubMed search was completed for relevant studies published between 2013 and 2023. Table 1 includes our complete search term used to conduct this analysis. Following this comprehensive search, two independent reviewers (ASO and MCM) screened all titles and abstracts. Articles including content related to our initial search strategy were selected for independent full-text review by the same two reviewers. Inclusion and exclusion criteria for final study selection are outlined in Table 1. A third reviewer (JME) resolved any disagreements regarding study inclusion. Author and reference tracking were conducted using selected studies to identify any relevant articles that were missing in our original search. Figure 2 outlines a complete flow diagram for study selection and inclusion.

**Table 1:** The PubMed search criteria and inclusion/exclusion criteria for the selected studies

Search Term	
*Total Joint Arthroplasty* **OR** *Total Hip Arthroplasty* **OR** *Total Knee Arthroplasty* **AND** *Smoking* **AND** *American College of Surgeons National Surgical Quality Improvement Program* **OR** *National Surgical Quality Improvement Program* **OR** *NSQIP* **AND** *Outcomes* **OR** *Complications*	
**Inclusion Criteria**	**Exclusion Criteria**
• Discusses primary and/or revision total hip and/or knee arthroplasty	• Discusses shoulder, ankle, metacarpal, or other non-hip or knee joint arthroplasty
• Includes theme of smoking as a specific predictor for selected outcomes	• Discusses machine learning or use of technology
• Utilizes ACS-NSQIP database	• Includes non-observational study designs
• Includes clearly defined variables and outcomes	• Includes risk calculator
• Published between 2013 and 2023	• Includes trend studies
• Has been cited in other publications	• Includes only a single outcome

### Data acquisition and analysis

The exact data from selected studies were obtained from our institution’s NSQIP office. Once obtained, data were analyzed by our statistician (ASO) utilizing identical statistical approaches employed by the original authors as defined in their methodology section. In cases where a required step was not explicitly stated in the original publication, the statistician made informed decisions to reproduce those steps (see Supplemental Table 1). Reproducibility was assessed by comparing the adjusted odds ratios (aORs) and their associated *p*-values (with α = 0.05) between the original paper results and the reanalyzed results. All data analyses were performed using SAS version 9.4 (TS1M1) (SAS Institute, Cary, North Carolina) [25].

## Results

Our initial literature search yielded 43 studies. Upon title and abstract screening, 36 studies were selected for full-text review. After full-text review, 10 studies met the inclusion criteria and 1 additional study was added via author/reference tracking for a net total of 11 included studies, all of which were observational studies (i.e. Level III evidence) (Fig. 2). Six studies evaluated smoking as a potential risk factor for postoperative complications after total joint arthroplasty. The remaining five studies assessed smoking and other potential risk factors associated with perioperative complications, increased length of hospital stay, and/or readmission rates after total joint arthroplasty (Table 2).

**Table 2:** Reproducibility of database studies in arthroplasty: a comparative analysis of original and reanalyzed data from ACS-NSQIP database

Study Number	Authors	Journal of Publication	Original Manuscript Findings	Reanalyzed Data Findings
1	Duchman et al., 2015	Journal of Bone and Joint Surgery	Current smokers have an increased risk of wound complications and both current and former smokers have an increased risk of total complications following total hip or total knee arthroplasty. Increasing pack-year history of smoking resulted in increasing total complication risk.	Current smokers have an increased risk of wound complications but did not have an increased risk of total complications following total hip or total knee arthroplasty. Former smokers have a reduced risk of total complications. There is no significant association between increasing pack-year history of smoking and total complications.
2	Liodakis et al., 2015	The Journal of Arthroplasty	Patients who underwent revision hip arthroplasty (RHA) had more perioperative complications, longer operative time, more blood transfusions, and longer hospital stay compared to those who underwent revision knee arthroplasty (RKA). The strongest modifiable risk factor for major complications and prolonged hospital stay after RHA and RKA was low preoperative hematocrit.	The reanalyzed data findings and conclusions were comparable to the original manuscript.
3	Keswani et al., 2015	The Journal of Arthroplasty	Multivariate analysis revealed risk factors for readmission after revision hip and knee arthroplasty including male sex, pulmonary disease, severe adverse event before discharge, stroke, cardiac disease, and American Society of Anesthesiologists (ASA) class 3 or 4. Independent predictors of extended length of stay included infection or fracture etiology relative to mechanical loosening etiology, dependent functional status, BMI ≥ 40 kg/m^2^, history of smoking, diabetes, cardiac disease, stroke, bleeding disorders, wound class 3 or 4, and ASA class 3 or 4.	Pulmonary disease is not a significant predictor for readmission after revision hip and knee arthroplasty. Fracture etiology relative to mechanical loosening etiology, history of smoking, and bleeding disorders were not significant predictors for extended length of stay in the revision knee arthroplasty group. Results were otherwise similar to the original manuscript.
4	Courtney et al., 2016	The Journal of Arthroplasty	Patients age ≥ 70 years, those with a cardiac history, smoking history, malnutrition, or diabetes have greater risk for readmission and complications after total joint arthroplasty (TJA). Outpatient TJA alone did not increase risk of readmission or reoperation, and it was a negative independent risk factor for postoperative complications.	Cardiac history is not a significant risk factor associated with readmission and complications after TJA. Results were otherwise similar to the original manuscript.
5	Sher et al., 2016	The Journal of Arthroplasty	Patients discharged within 24 h after total joint arthroplasty (TJA) were more likely to be younger, male sex, American Society of Anesthesiologists (ASA) class 1 or 2, and less likely to be taking steroids or have comorbidities. Multivariate analysis revealed independent predictors for adverse events or readmission including age ≥ 80 years, smoking, bleeding disorders, ASA class 3 or 4, and experiencing severe adverse events (SAE) prior to discharge.	Smoking, ASA class 3 or 4, and experiencing SAE prior to discharge were not significant predictors for adverse events or readmission after TJA. Results were otherwise similar to the original manuscript.
6	Bedard et al., 2018	The Journal of Arthroplasty	Multivariate analysis showed that current smokers have an increased risk of deep infection and reoperation after revision total hip arthroplasty (THA). Smoking status additionally had no effect on wound complications after revision THA.	Current smokers did not have an increased risk of deep infection or reoperation after revision THA. Results were otherwise similar to the original manuscript.
7	Bedard et al., 2018	The Journal of Arthroplasty	Multivariate analysis showed that current smokers have an increased risk of any wound complication and deep infection after revision total knee arthroplasty (TKA). Smoking status additionally had no effect on reoperation after revision TKA.	Current smokers have an increased risk of any wound complication; however, current smokers did not have an increased risk of deep infection after revision TKA. Results were otherwise similar to the original manuscript.
8	Sahota et al., 2018	The Journal of Arthroplasty	Smokers in the combined total hip and knee arthroplasty cohort had higher rates of readmission and deep surgical site infection compared to non-smokers. Smokers in the combined cohort were also more likely to have a surgical complication compared to non-smokers.	The reanalyzed data findings and conclusions were similar to the original manuscript.
9	Johnson et al., 2019	The Journal of Arthroplasty	Increasing age, obesity, smoking, diabetes, chronic obstructive pulmonary disease (COPD), hypertension, bleeding disorders, corticosteroid use, and dependent functional status conferred an increased risk of discharge after 24 h following total knee arthroplasty (TKA). Male gender, spinal anesthesia, and monitored anesthesia care were protective against length of stay greater than 24 h.	The reanalyzed data findings and conclusions were similar to the original manuscript.
10	Agrawal et al., 2021	The Journal of Arthroplasty	Multivariate analysis revealed smokers have an increase in pulmonary and infectious complications and longer hospital stays compared to non-smokers.	Smokers do not have an increase in pulmonary complications compared to non-smokers. Results were otherwise similar to the original manuscript.
11	Heckmann et al., 2021	Orthopedics	Risk of total complications or thrombotic events is not accentuated in smokers who underwent total joint arthroplasty. Regardless of pack-year exposure, smokers have increased risk of readmission and wound complications.	The reanalyzed data findings and conclusions were similar to the original manuscript.

Among the 11 reanalyzed studies, a total of 268 aORs were examined. Of these 268 original aORs, 34 aORs (12.69%) changed in interpretation from harmful to protective or vice versa upon reanalysis and 36 aORs (13.43%) experienced a change in statistical significance (Table 3). Furthermore, the average magnitude change for each individual aOR was 17.22% (range = 2.10 − 47%; median = 13.67%) across included studies, and the total sample size (N) of included studies varied by an average of 2.83% (range = 0 − 47.84%; median = 5.75%). Seven studies had conclusions that changed in this reanalysis, with approximately one in eight objective conclusions varying in interpretation or statistical significance. Table 2 outlines the original conclusions of selected studies and how these findings changed upon reanalysis.

**Table 3:** Summary of reproduced adjusted odds ratios (aORs). Specifically, reproduced aORs that changed in interpretation from the original study, changes in statistical significance in reproduced aORs, and changes in sample size between original and reproduced data are included

Study Number	aORs				Statistical Significance*		Comparison of Sample Size (*N*)				
	Total aORs in Study	Reproduced aORs that changed in interpretation	Total aOR Change (%)	Average Magnitude Change of aOR (%)	Reproduced aORs that changed in significance	Significance Change (%)	Original Total (*N*)	Reproduced Total (*N*)	Difference in Sample Size	Difference in Sample Size (%)	
1	15	4	26.67	9.40	5	33.33	78,191	83,736	5545	7.09	
2	42	3	7.14	2.10	6	14.29	5068	5198	130	2.57	
3	56	2	3.57	26.34	5	8.93	10,112	9788	324	3.20	
4	40	5	12.50	17.01	5	12.50	169,406	169,406	0	0.00	
5	35	4	11.43	39.49	1	2.86	120,742	167,402	46,660	38.64	
6	3	0	0.00	17.30	2	66.67	8237	8790	553	6.71	
7	3	0	0.00	13.67	1	33.33	8776	7945	831	9.47	
8	3	0	0.00	47.00	2	66.67	2502	2476	26	1.04	
9	24	0	0.00	6.79	0	0.00	210,075	210,064	11	0.01	
10	7	2	28.57	4.99	5	71.43	67,897	35,413	32,484	47.84	
11	40	14	35.00	5.35	4	10.00	2088	2208	120	5.75	
**Total**	**268**	**34**	**12.69%**	**17.22%**	**36**	**13.43%**	**683,094**	**702,426**	**19,332**	**2.83%**	

*Determined by *p* < 0.05

## Discussion

Observational research utilizing large clinical registries represents a growing area of orthopaedic literature. However, concerns exist regarding inadequate reporting of study methodology in many of these orthopaedic studies [22]. Despite these concerns, the reproducibility of such studies has not been adequately assessed in existing literature. This study aimed to determine the reproducibility of ACS-NSQIP studies on smoking as a risk factor for poor surgical outcomes after total joint arthroplasty. Our results demonstrated inconsistent reproducibility in many of these studies, with significant variability observed in included sample size, calculation of aORs, and study conclusions despite employing identical datasets and statistical methods. These findings highlight the importance of rigorous reporting of study methodology, data collection, and statistical analyses when utilizing large-volume databases in orthopaedic research.

Recent studies have highlighted that most observational database research has inadequate reporting of study methodology. Khera et al. analyzed 120 studies published between 2015 and 2016 using the National Inpatient Sample (NIS) database in both medical and surgical fields and discovered that most did not adhere to recommended practices for methodologic reporting [21]. In orthopaedics specifically, Teng et al. evaluated 136 studies published between 2016 and 2017 using the NIS and found again that the majority did not adhere to recommended practices for reporting methodology [22]. Insufficient detailing of these methods may cast doubt on the integrity and conclusions of the study, particularly when attempts to reproduce findings based on the reported methodology yield divergent results.

Several features inherent to this type of research may predispose to these shortcomings. The complexity and vastness of data collected from these sources makes it challenging to provide both succinct and comprehensive descriptions of methodologic practices [26]. The extensive process of data extraction, handling, and cleaning prior to statistical analysis can also hinder researchers’ ability to recall and articulate intricate details of their methods accurately [27]. These intricacies of data handling, such as how missing values were addressed within the study, can lead to substantial differences in sample size or findings during methodologic appraisal or study reproduction if not reported correctly. Stringent word count limitations often imposed by journals compound these challenges, compelling authors to abbreviate or omit detailed descriptions of methodology in order to prioritize the results and discussion sections. [23]. This practice errantly assumes that readers will grasp and trust study methodology implicitly by virtue of the manuscript undergoing peer review; however, peer review alone does not guarantee study reliability and validity, and readers should be given the opportunity to assess this independently. The absence of standardized reporting guidelines for methodology in many orthopaedic journals also contributes to these inconsistencies, resulting in further variation in reporting of study methods in observational database research [22].

Given the utility of these study designs, focused efforts on addressing transparency, clarity, and adherence to rigorous reporting standards are needed to enhance the reliability of orthopaedic database research. From an author perspective, researchers must ensure to employ high standards for reporting by providing rigorous detail of methodologic practices that enables accurate assessment of study validity and reproducibility. This must be done with specific attention towards outlining: (1) the database utilized and its potential applications, strengths, and limitations in the study, (2) protocols for data extraction and handling of any missing values or duplicate records in the initial dataset, (3) data handling and cleaning practices to achieve the final dataset, and (4) any subsequent analyses performed with the resulting data [28]. In instances where limited word count hinders the ability to document this information, authors should take advantage of the use of tables, figures, supplemental materials, and/or appendices to provide sufficiently detailed descriptions of methodology [24]. Utilization of trained statisticians or health sciences writing experts at institutional libraries may also provide aid in writing this section with an appropriate level of detail [29]. From a reviewer and editor perspective, thorough methodologic appraisal is critical to ensure studies have sufficient reporting prior to their publication. Utilization of standardized methodologic reporting guidelines, such as the Strengthening the Reporting of Observational Studies in Epidemiology (STROBE) checklist, by orthopaedic journals may improve adherence rates while also making the job of reviewers and editors easier [22, 30]. From a broader journal perspective, expanding or eliminating word count limitations in journals may provide additional space to allow adequate reporting of study methods. Furthermore, demanding raw data from the final dataset in addition to reproducible, step-by-step instructions for how this final dataset was achieved may provide further transparency and methodologic integrity in observational database research; however, this must be balanced with a concern for patient confidentiality and privacy in the provided datasets [23].

### Limitations

This study is not without limitations. First, this study limited our initial search to PubMed exclusively, potentially excluding articles of interest that are not indexed in PubMed. Author and reference tracking was incorporated into our comprehensive search strategy to mitigate this unintended consequence and reduce the likelihood of article exclusion. Second, our study focused solely on manuscripts utilizing the ACS-NSQIP database. Numerous clinical registries exist and have been used in orthopaedic literature, and inconsistent reproducibility may have arisen from inherent features of NSQIP database. However, limiting the scope of our analysis to studies using this database helped facilitate detailed reproduction of study methodology while also reducing confounding due to variability between different clinical registries. Third, some degree of variability in study reproduction may be due to intrinsic features of the NSQIP database, such as differences in hospital participation, data updates, or data retrieval processes over time. Lastly, our study only included manuscripts in arthroplasty focusing on smoking as a risk factor for poor surgical outcomes. While this represents only a subset of orthopaedic literature and may reduce generalizability to the broader field, this remains one of the most common applications of these registries in orthopaedic surgery research. Narrowing our focus in this manner also enabled thorough reanalysis of manuscript findings within this specific area of research. Further studies should be conducted to assess the reproducibility of observational database research across other orthopaedic subspecialties.

## Conclusion

Utilization of large-volume clinical registries for observational research has gained popularity in orthopaedic literature. However, the validity and reproducibility of such studies is often overlooked. This study aimed to determine the reproducibility of ACS-NSQIP studies evaluating smoking as a risk factor for poor surgical outcomes after total joint arthroplasty. Our results indicate that reproducibility of many of these studies is inconsistent, with significant variability observed in included sample size, calculation of aORs, and study conclusions despite employing identical datasets and statistical methods. These findings highlight the importance of rigorous reporting of study methodology, data collection, and statistical analyses when utilizing large-volume databases in orthopaedic research. This burden of responsibility should be shared among authors, peer reviewers, and orthopaedic journals to confirm the accuracy and validity of published database research.

## Electronic supplementary material

Below is the link to the electronic supplementary material.

Supplementary Material 1

## Data Availability

The data supporting the conclusions of this article are available in the American College of Surgeons National Surgical Quality Improvement Program (ACS-NSQIP) repository, [https://www.facs.org/quality-programs/data-and-registries/acs-nsqip/].

## Abbreviations

ACS-NSQIP: American College of Surgeons National Surgical Quality Improvement Program
aOR: Adjusted odds ratio
NIS: National Inpatient Sample
STROBE: Strengthening the Reporting of Observational Studies in Epidemiology
