# Supplementary material for: Assessing the reproducibility of American College of Surgeons National Surgical Quality Improvement Program (ACS-NSQIP) arthroplasty studies
Source: J Orthop Surg Res. 2025 Mar 1;20:216. doi: 10.1186/s13018-025-05538-0 (PMC11871755; doi:10.1186/s13018-025-05538-0)
Supplement: Supplementary file 1 — Supplementary Material 1 [file 13018_2025_5538_MOESM1_ESM.docx]

1. **Duchman et al, 2015**

**Journal: Journal of Bone and Joint Surgery**

This manuscript did not discuss key aspects of data cleaning such as how extreme outliers/implausible values, duplicate records, or missing data were handled during analysis. In our re-analysis, we approached data cleaning systematically to ensure transparency and reproducibility:

- Outliers or Implausible Values: Extreme outliers and implausible values, identified based on clinical relevance or statistical measures (e.g., interquartile range method), were excluded.
- Duplicates: Duplicate records were identified and removed to prevent inflation of sample size.
- Missing Data: Records with more than 40% missing variables were excluded from the analysis. For records with less than 40% missing data, imputation was not applied, and those variables were omitted from statistical models if necessary.

1. **Liodakis et al, 2015**

**Journal: The Journal of Arthroplasty**

This manuscript did not specify how much missing baseline data was tolerated in analysis. In our re-analysis, records with more than 40% missing baseline data were excluded as specified above.

1. **Keswani et al, 2015**

**Journal: The Journal of Arthroplasty**

This manuscript did not describe how duplicate records were handled, as well has how incomplete data were handled during analysis. In our re-analysis, duplicate records were removed and records with more than 40% missing data were excluded as specified above.

1. **Courtney et al, 2016**

**Journal: The Journal of Arthroplasty**

This manuscript had discrepancies observed in the number of certain variables including smoking history, return to the operating room, malnourished status, and mortality. For example, despite using the same criteria for malnourished as the original study (i.e. preoperative albumin levels below 3.5 g/dL), different sample numbers were identified in re-analysis. The original study additionally did not specify how duplicate records were handled. In our study, re-analysis was performed using the same criteria as specified in the original manuscript. Duplicate records were additionally removed from re-analysis as specified above.

1. **Sher et al, 2016**

**Journal: The Journal of Arthroplasty**

This manuscript did not specify key data cleaning steps including how duplicate records were handled. Additionally, variables contributing to “severe complications” were not fully described, and Table 3 did not provide sufficient detail to provide direct comparison. In our re-analysis, duplicate records were removed as specified above and attempts to clarify ambiguous variables were made as best as possible.

1. **Bedard et al, 2018 (THA)**

**Journal: The Journal of Arthroplasty**

This manuscript did not outline how extreme outliers and duplicate records were handled during analysis. Additionally, the description of the multivariate model did not specify whether variables such as age and BMI were treated as continuous or categorical variables, introducing uncertainty that could influence the results/interpretation of the model. In our re-analysis, extreme outliers/implausible values were excluded and duplicate records were removed as specified above. Age and BMI were treated as continuous variables in reproduction of the multivariate model.

1. **Bedard et al, 2018 (TKA)**

**Journal: The Journal of Arthroplasty**

This manuscript did not specify how extreme outliers and duplicate records were handled during analysis. In our re-analysis, extreme outliers/implausible values were excluded and duplicate records were removed as specified above.

1. **Sahota et al, 2018**

**Journal: The Journal of Arthroplasty**

This manuscript did not provide specific details regarding the caliper size used for propensity score matching. In our re-analysis, we utilized default settings of our statistical program for caliper size. Please note, our re-analysis utilized SAS whereas the original study utilized SPSS, which may account for minor differences in the matched cohorts due to variations in default settings or implementation of matching algorithms.

1. **Johnson et al, 2019**

**Journal: The Journal of Arthroplasty**

This manuscript did not specify how duplicate records were handled during analysis. In our re-analysis, duplicate records were removed as specified above.

1. **Agrawal et al, 2021**

**Journal: The Journal of Arthroplasty**

This manuscript did not specify how duplicate records were handled during analysis. In our re-analysis, duplicate records were removed as specified above.

1. **Heckmann et al, 2021**

**Journal: Orthopedics**

This manuscript did not specify what statistical software was used for analysis. In our study, SAS was utilized to complete re-analysis with our data findings and conclusions yielding similar overall results as the original study.

**Supplemental Table 1**: Summary of included studies, details of analytical methods that were not specified in the original study, and our statistician’s approach during re-analysis.

| **Study** | **Details Not Specified** | **Approach Taken in Reanalysis** |
| --- | --- | --- |
| Duchman et al., 2015 | Data cleaning steps including handling of outliers, duplicate records, and missing values. | Excluded cases with >40% missing data, removed duplicates, excluded outliers and implausible values. |
| Liodakis et al., 2015 | How much missing baseline data was tolerated within exclusion criteria. | Excluded cases with >40% missing baseline data. |
| Keswani et al., 2015 | Handling of duplicate records and incomplete data. | Removed duplicates, excluded cases with >40% missing data. |
| Courtney et al., 2016 | Unclear variable definitions with discrepancies observed in number of certain variables, handling of duplicate records. | Utilized variable definitions as specified in original study, removed duplicates. |
| Sher et al., 2016 | Handling of duplicate records, unclear variable definitions for "severe complications". | Removed duplicates, clarified ambiguous variables as best as possible. |
| Bedard et al., 2018 (THA) | Handling of outliers and duplicate records. Details of treatment of variables in multivariate model. | Excluded outliers and implausible values, removed duplicates. Treated age/BMI as continuous variables in multivariate model. |
| Bedard et al., 2018 (TKA) | Handling of outliers and duplicate records. | Excluded outliers and implausible values, removed duplicates. |
| Sahota et al., 2018 | Details of Caliper size used for propensity score matching. | Utilized default settings for caliper size in our statistical program. Noted statistical software differences (SAS versus SPSS). |
| Johnson et al., 2019 | Handling of duplicate records | Removed duplicates |
| Agrawal et al., 2021 | Handling of duplicate records | Removed duplicates |
| Heckmann et al., 2021 | Statistical software used for analysis. | Conducted analysis using SAS, noted potential difference in statistical software utilized. |
